# The reliability and validity of the Survey of Activities and Fear of Falling in the Elderly for assessing fear and activity avoidance among stroke survivors

**DOI:** 10.1371/journal.pone.0214796

**Published:** 2019-04-01

**Authors:** Tai-Wa Liu, Shamay S. M. Ng

**Affiliations:** 1 Department of Rehabilitation Sciences, The Hong Kong Polytechnic University, Hung Hom, Hong Kong (SAR); 2 School of Nursing and Health Studies, The Open University of Hong Kong, Ho Man Tin, Hong Kong (SAR); University of Malaya, MALAYSIA

## Abstract

**Introduction:**

Activity restriction due to fear of falling is a common problem after a stroke. It can lead to deteriorated physical condition, restricted social participation and deprived quality of life. The Survey of Activities and Fear of Falling in the Elderly (SAFE) was developed to assess these difficulties, and its utility has been demonstrated among the older adults and older people with Parkinson’s disease. This study aimed to expand those demonstrations to community-dwelling chronic stroke survivors using a Chinese translation of the instrument.

**Methods:**

One hundred and eight elderly individuals with a history of stroke completed the Chinese version of the SAFE (SAFE-C). The internal consistency of their responses was assessed using Cronbach’s alpha. Twenty of the same subjects were reassessed after a 1-week interval to assess the instrument’s test-retest reliability. Structure validity was examined by exploratory factor analysis. Pearson correlation was used to establish the instrument’s convergent validity with respect to the results from the Chinese version of the Activities-specific Balance Confidence Scale (ABC-C) and the Chinese version of the Lawton Instrumental Activities of Daily Living scale (IADL-C).

**Results:**

The items of the SAFE-C demonstrated excellent internal consistency with a Cronbach’s alphas of 0.90. The SAFE-C also had excellent test-retest reliability with an overall intra-class correlaton coefficient of 0.91. A 1-factor structure termed “fear avoidance circumstances” was identified and it was shown to be consistent with the original measure developed among community-living older people. The correlations between the SAFE-C and the ABC-C (r = -0.68) and the IADL-C (r = -0.57) confirmed the convergent validity.

**Conclusions:**

Fear avoidance behavior is a homogeneous construct applicable to people with stroke-specific impairments alike. The SAFE-C is a reliable and valid instrument for measuring the level of fear avoidance behavior among community-dwelling stroke survivors. Stroke survivors with good functional mobility revealed a low level of fear and avoidance as measured by the SAFE-C.

## Introduction

Fear of falling is a common psychological trait among stroke survivors, with self-reported prevalence rate high up to 49% among those resided in the community [1]. The major adverse consequence is that fear of falling can be reflected in the form of self-imposed restrictions on physical activity. That can of course lead to various negative health outcomes such as physical deconditioning [2], decreased competence in the activities of daily living [3], limited social participation [4] and eventually a degraded quality of life [5]. Fear of falling may not necessarily restrict activity, however [6, 7]. A study by Howland [7] found that 55% of 266 community-dwelling elderly adults reported fear of falling, but only 56% of them reported associated activity curtailment. Advanced age, female gender, impaired vision, poor perceived health, lack of support and a history of multiple falls are risk factors for activity restriction due to fear of falling [3,8].

Despite fear avoidance behavior having been studied among community-dwelling healthy older adults, most previous studies [6, 9] have used questions such as “Do you avoid certain activities due to concerns about falling” [9] or “Has fear of falling made you avoid any activities?” [6] to classify people as with or without activity restriction due to fear of falling. The use of a single dichotomous question makes for easy categorization for research purposes, but it provides insufficient information for clinicians to address rehabilitation needs. Lachman [10] developed the Survey of Activities and Fear of Falling in the Elderly (SAFE) intended to evaluate fear avoidance behavior due to fear of falling. The SAFE assesses the level of activity restriction through quantifying self-perceived and observable activities of daily living (ADL) and instrumental ADL (IADL). The original SAFE consisted of 11 items rated on a 4-point scale. It was validated with 270 healthy community-dwelling older people and demonstrated excellent internal consistency (Cronbach’s alpha = 0.91) and convergent validity with the Falls Efficacy scale (FES) (r = -0.76). It has also demonstrated criterion validity with quality of life as measured by the Medical Outcome Study Short Form 36 (SF-36) (r = -0.37 to 0.55) [11] and discriminant validity in distinguishing those with or without fear of falling [10]. The Persian version of the SAFE [12] has been shown to have excellent test-retest reliability (Intra-class correlation coefficient = 0.97), and good correlation with Activities-specific Balance Confidence (ABC) scale ratings [13] (r = -0.87) among older people with Parkinsonism. The SAFE has been translated into Chinese (as the SAFE-C) and has been reported to have excellent internal consistency (Cronbach’s alpha = 0.95) with older people with depression symptoms [14].

Although improving functional status is an important focus of stroke rehabilitation, activity restriction can jeopardize clinicians’ efforts, as fear avoidance behavior may hinder stroke survivors’ performing physical and independent living activities. Despite the fact that the SAFE has been used with community-dwelling older people and elderly people with Parkinson’s disease, its psychometric properties have not been established for stroke survivors. This study was therefore designed to validate the SAFE among community-dwelling people after a stroke. Its objectives included investigating the instrument’s internal consistency, test-retest reliability, convergent validity and construct validity as well as the level of fear avoidance behavior manifested by community-dwelling stroke survivors.

## Materials and methods

### Participants

In total, 108 community-dwelling stroke survivors were recruited from a local rehabilitation organization in Hong Kong through poster advertising. Their demographics are summarized in Table 1. Subjects were recruited if they were at least 50 years old, had suffered a single stroke confirmed by the magnetic resonance imaging or computed tomography at least 1 year before the start of the study, were able to rise from a chair without using any arm support, and had an Abbreviated Mental Test [15] score of 7 or above. Subjects were excluded if they had any other medical condition that potentially could affect the findings such as chronic knee pain. The Departmental Research Committee of the Hong Kong Polytechnic University has approved the study. Written informed consent was obtained from all of the participants before the study.

**Table 1:** Characteristics of the subjects (n = 108).

Characteristics		Value, mean ± SD (range)
Age		60.37 ± 6.30 (50–78)
Gender, no.		
	Male/Female	71/37
Education, no.		
	< Secondary school	23
	Secondary school	68
	College or above	17
Living, no.		
	Alone	6
	With family/carer/friend	102
Employment, no.		
	Full time	3
	Part time	6
	Retired	53
	Unemployed	46
BMI (kg/m^2^)		24.19 ± 3.13 (15.96–32.65)
Cause of stroke, no.		
	Ischemic	46
	Hemorrhagic	62
Hemiplegic side, no.		
	Left/Right	55/53
Years since stroke, year		7.64 ± 4.39 (1.65–16.90)
No. of falls in past 6 months, no.		
	0	85
	1	12
	≥ 2	11
Mobility status, no.		
	Unaided	27
	Stick	52
	SBQ	15
	LBQ	5
	Others	9

BMI, body mass index; SBQ, small base quadripod; LBQ, large base quadripod.

### Outcome measures

All 108 participants were assessed with three questionnaires through an individual face-to-face interview in a university-based neurorehabilitation laboratory during April to May 2018. 20 of the subjects (19%) were randomly selected by drawing lots for reassessment with SAFE-C after one week to establish test-retest reliability.

#### SAFE-C score

The current SAFE [10] has 11 self-rated items with a one-factor structure assessing the level of fear of falling while performing activities such as “go to the store” and “take a tub bath”. There are four response options (0 = not all worried; 1 = a little worried; 2 = somewhat worried; 3 = very worried). To assess the level of activity avoidance due to fear of falling, the SAFE uses structured interviewing questions (e.g., “When you go to the store, how worried are you that you might fall?”; “Do you not go to the store because you are very worried that you might fall?” according to the scoring information guide. The Chinese version (SAFE-C) [14] is a translation of the original version in which 22 items were included. The SAFE-C had been demonstrated to have excellent internal consistency 0.95 (Cronbach’s alpha = 0.95) [14].

#### Activities-specific balance confidence score

Fear of falling was also measured using the ABC-C [16], a Chinese translation of the 16-item Activities Balance Confidence Scale. Respondents rate indoor and outdoor functional activities from 0% (no confidence) to 100% (complete confidence) representing their subjective confidence about maintaining their balance. The ABC-C [16] has been used with healthy older people and those with chronic disease such as stroke. It had been demonstrated to have excellent internal consistency of 0.97 (Cronbach’s alpha = 0.97) [16]. As the FES, which was adopted in the development study of original SAFE, focused on assessing the balance efficacy related to indoor activities only, this study adopted the ABC-C to assess the fear of falling related to both indoor and outdoor activities.

#### Lawton instrumental activities of daily living score

Level of independence in daily living was assessed using the IADL-C [17], a Chinese translation of the Lawton Instrumental Activities of Daily Living questionnaire. Respondents rate their perceptions of their independence in 9 daily household or community functions using a 3-point Likert scale (0 = unable to do, 1 = with assistance, 2 = independent). The IADL-C has been validated with institutionalized older adults and reported to have good internal consistency (Cronbach’s alpha = 0.86) and excellent inter-rater (ICC = 0.99) and test-retest (ICC = 0.90) reliability [17].

### Data analysis

The data were analyzed using version 23.0 of the SPSS software suite (IBM, Armonk, NY). The level of confidence for significance was set as α = 0.05. Internal consistency was evaluated using Cronbach’s alpha coefficient. Test-retest reliability was assessed using intra-class correlation coefficients (ICCs). An ICC >0.90 indicates excellent reliability, an ICC of 0.75 to 0.90 indicates good reliability, an ICC of 0.50 to 0.75 indicates moderate reliability, and an ICC <0.50 indicates poor reliability [18]. To establish the optimal factor structure for the SAFE-C, principle component analysis (PCA) was performed and scree plots were prepared. Principal axis factoring (PAF) was used for data extraction, followed by promax rotation to enhance the interpretability of the factors. Pearson correlation coefficients were used to establish the convergent validity of the SAFE-C with the ABC-C and IADL-C results. The strength of the correlation was defined as weak (< 0.35), moderate (0.36 to 0.67), or strong (0.68 to 1.0) [19].

## Results

The assessments were performed in a university-affiliated neurorehabilitation laboratory. After providing written consent, the participants completed a demographic data sheet and the ABC-C and IADL-C. Then the participants were interviewed for SAFE-C according to the scoring information guide.

### Characteristics of the subjects

Most of the 108 respondents were men (n = 71, 66%) with educational attainment up to secondary school level (n = 68, 63%) (see Table 1). Almost all (n = 102, 94%) were living with family, a carer or a friend and were unemployed or retired (n = 99, 92%). Most had not fallen in the 6 months before the study (n = 85, 79%). They had mean a body mass index (BMI) of 24.19 (SD 3.13) and 81 (75%) of them relied on a walking aid when walking outdoors. Their mean age was 60.37 years (SD 6.30) and the mean years since stroke was 7.64 years (SD 4.39).

The mean scores of all the SAFE-C items and their standard deviations are summarized in Table 2. The total mean score was 9.75 (SD 7.21, range from 0 to 28). Item 6 (Go out when slippery) had the highest average (1.44 ± 0.95) and item 4 (Get out of bed) the lowest (0.36 ± 0.72).

**Table 2:** Mean and standard deviation (SD) of SAFE–C item scores.

Items	Mean	SD
1. Go to store	0.84	0.93
2. Prepare simple meals	0.65	0.90
3. Take a tub bath	1.30	1.06
4. Get out of bed	0.36	0.72
5. Take a walk for exercise	0.60	0.85
6. Go out when slippery	1.44	0.95
7. Visit a friend or relative	0.73	0.90
8. Reach over head	0.98	0.94
9. Go to place with crowds	1.35	0.99
10. Walk several blocks outside	0.79	1.01
11. Bend down	0.71	0.95
Total score	9.75	7.21

### Internal consistency

Taken together, the items of the SAFE-C had excellent internal consistency with a Cronbach’s alpha of 0.90 (see Table 3). There was moderate to strong item-total correlation ranging from 0.47 to 0.72. Although four of the item-total correlations were < 0.60 (Go to the store, r = 0.59; take a tub bath, r = 0.53; get out of bed, r = 0.47; take a walk for exercise, r = 0.54), there was no item who’s deletion improved the overall Cronbach’s alpha.

**Table 3:** Internal consistency of the SAFE–C.

Item No.	Item	Corrected Item-Total Correlation	Alpha if Item Deleted
1.	Go to the store	0.59	0.89
2.	Prepare simple meals	0.67	0.89
3.	Take a tub bath	0.53	0.90
4.	Get out of bed	0.47	0.90
5.	Take a walk for exercise	0.54	0.89
6.	Go out when slippery	0.65	0.89
7.	Visit a friend or relative	0.67	0.89
8.	Reach over head	0.72	0.88
9.	Go to place with crowds	0.71	0.88
10	Walk several blocks outside	0.70	0.89
11	Bend down to get something	0.70	0.89

NOTE. Cronbach’s α coefficient for the entire SAFE-C is 0.90.

### Test-retest reliability

Twenty of the 108 respondents were reassessed after a 1-week interval to quantify the SAFE-C’s test-retest reliability. The total SAFE-C score had excellent test-retest reliability after a week as reflected in an ICC of 0.91 (95% confidence interval (CI) 0.76–0.96) (see Table 4). Test-retest correlation coefficients for individual items ranged from 0.52 to 0.93, with item 9 (Go to a place with crowds, ICC = 0.93 95% CI 0.83–0.97) indicating the most consistency, and item 1 (Go to the store, ICC = 0.52 95% CI 0.22–0.81) the least. Overall, the 0.91 ICC indicated good reproducibility for SAFE-C ratings over time.

**Table 4:** Test-retest reliability of the SAFE–C (n = 20).

Test-retest reliability					
	Mean Score			95% CI	
Item	1^st^	2^nd^	ICC	Lower	Upper
1.	0.55	0.25	0.52	-0.22	0.81
2.	0.30	0.40	0.80	0.48	0.92
3.	1.25	0.95	0.79	0.47	0.92
4.	0.25	0.15	0.92	0.80	0.97
5.	0.25	0.20	0.73	0.32	0.89
6.	1.20	1.20	0.87	0.67	0.95
7.	0.50	0.45	0.81	0.53	0.93
8.	0.85	0.90	0.90	0.74	0.96
9.	1.25	1.20	0.93	0.83	0.97
10.	0.40	0.30	0.84	0.60	0.94
11.	0.60	0.60	0.84	0.59	0.94
Total SAFE–C score	7.40	6.60	0.91	0.76	0.96

### Convergent validity

The correlation coefficient relating the SAFE-C and ABC-C results was r = -0.68 (95% CI -0.82 to -0.54), and that relating the SAFE-C and IADL-C results was r = -0.57 (95% CI -0.73 to -0.41). There was moderate to strong negative correlation between the pairs of measures. Both the correlations were statistically significant (*p*≤0.001).

### Construct validity

The PCA suggested a 2-factor structure accounting for 54% of the total variance in which each factor with eigenvalue exceeding 1. The Kaiser-Meyer-Olkin (KMO) value of 0.89 indicated sampling adequacy [20]. Bartlett’s test was highly significant (*X*^2^ (55) = 569.30, *p*≤0.001) suggesting an appropriate use of factor analysis [21]. Examination of the scree plot suggested that there were 2 or 3 factors above the break point of the data. To avoid under- or over-extraction of the number of factors, we also consider +/- 1 number from the numbers suggested by the scree plot and PCA. Therefore, the numbers of factors used for extraction were 1, 2, 3, and 4. After promax rotation, the loadings of all 11 items were greater than 0.30 [22] on the 1-factor structure, ranging from 0.49 to 0.76 with an eigenvalue of 5.50 and 45.2% of the total variance explained (see Table 5). Although the 2, 3 and 4 factor structures could explain more of the variance, they all have item crossloadings on more than 1 factor. Thus, the 1-factor structure was adopted.

**Table 5:** Rotated factor matrix of the SAFE-C based on the principal axis factoring with promax Rotation.

Item		1-factor
(8)	Reach over head	0.76
(9)	Go to place with crowds	0.75
(10)	Walk several blocks outside	0.75
(11)	Bend down	0.74
(2)	Prepare simple meals	0.71
(7)	Visit a friend or relative	0.71
(6)	Go out when slippery	0.69
(1)	Go to store	0.62
(5)	Take a walk for exercise	0.57
(3)	Take a tub bath	0.56
(4)	Get out of bed	0.49
	Eigenvalues	5.50
	Variance explained (%)	45.22

## Discussion

This is the first study designed to investigate the psychometric properties of the SAFE-C using people with chronic stroke. It revealed the excellent internal consistency and excellent test-retest reliability of the SAFE-C. It also revealed its concurrent validity, as it showed significant correlation with ABC-C and IADL-C results. The instrument’s key construct was identified factor as “fear avoidance circumstances” which takes in common community living activities in which falls usually occur. That 1-factor structure is consistent with those suggested by Lachman [10] for use with community-dwelling and healthy older adults.

Previous studies using dichotomous “yes/no” questions revealed that around 41% of healthy older adults had fear avoidance behavior [8]. The main limitations of using a dichotomous “yes/no” question are that it cannot (i) inform about the overall level of fear of falling during activities, (ii) reflect what kinds of physical activity are being restricted, and (iii) provide enough information to estimate whether or not the fear is excessive. A strength of this study is the use of a validated instrument to unravel which daily physical activities most influence fear of falling. The overall mean item score of 9.75 indicates significant activity restriction due to fear of falling. The 3 least fear-inducing daily activities—getting out of bed, taking a walk for exercise and preparing simple meals—are all necessary for wholesome independent living. The low level of activity restriction associated with those three items may indicate only minimal impact of fear of falling on self-care and on maintaining healthy habits.

On the other hand, the 3 most fearful daily activities—going out when it’s slippery, going to places with crowds and taking a tub bath—are indeed common fall settings with alternatives which allow them to be avoided. Recognizing the fall risks in these circumstances could be interpreted as wise wariness. It does not necessarily entail activity curtailment. After all, walking several blocks outside and go to the store induced minimal fear of falling. Thus, it is reasonable to infer that the study participants knew they had alternatives. If so, the findings could be interpreted as confirming that the avoidance measured is neither restrictive nor excessive, but rather protective instead.

The validation results reveal that the SAFE-C has excellent internal consistency (Cronbach’s alpha of 0.90) comparable with that of the original English SAFE (Cronbach’s alpha of 0.91). The SAFE-C has also been assessed with older adults showing symptoms of depression where it yielded a Cronbach’s alpha of 0.95. It can be inferred that activity restriction arising from fear of falling after a stroke was consistently measured across the 11 items of the SAFE-C.

The SAFE-C showed excellent test-retest reliability (ICC = 0.91, 95% CI 0.76–0.96), though it was slightly lower than that reported for the Persian version assessed using people with Parkinson’s disease (ICC = 0.97, minimum detectable change at the 95% confidence level of 5.28). The excellent test-retest reliability suggests that SAFE-C results are highly reproducible for people with stroke, which is a desirable feature for clinical use. Although item 1 (Go to the store) showed only fair consistency across the 2 measurements, the overall reliability of the measure was not impaired when item 1was retained in the instrument. In addition to real changes in the level of fear of falling while going to the store and its avoidance, several other reasons might contribute to the low repeatability of item 1. Self-perceptions of balance ability might for some reason be particularly inconsistent in the shopping context. Or there may have been inconsistent interpretation of the “go to the store” scenario.

Fear of falling is generally quite realistic for such subjects, so SAFE-C results should correlate with balance efficacy as measured by the ABC-C. Indeed, the data show a strong negative correlation (r = -0.68). A similar correlation was found among people with Parkinsonism (r = -0.87) [12] and among healthy older adults with poor balance as measured using the Falls Efficacy Scale (r = -0.76) [10].

The strength of correlation between the SAFE-C and IADL-C scores (r = -0.57) was relatively weaker than between the SAFE-C and ABC-C scores (r = -0.68). That would be explained if some of the subjects’ dependency in daily living was due to stroke-related physical impairments but not to fear of falling. Also, some of the subjects’ fear avoidance behavior was related to social activities, not to typical activities of daily living. It is thus possible that these subjects had little fear avoidance behavior but a high level of dependency, or vice versa. In any case, the correlation between the SAFE-C and the ABC-C results was not close to unity [21], which is consistent with the suggestions of previous studies [4, 7] that people with impaired balance may or may not be fearful and may or may not practice avoidance. The correlations between the SAFE-C, the ABC-C and the IADL-C results confirm their convergent validity.

This is the first study to examine the factor structure of the SAFE in population of patients with chronic disabling conditions. The hypothesized 1-factor structure based on fear and avoidance circumstances is consistent with those reported for the Chinese population in Hong Kong with chronic stroke. Although the factor loadings (0.49–0.76) were not similar to those found with healthy older adults in the original study (0.52–0.80) [10], they constitute a coherent factor structure supporting measurement of the construct among healthy older adults and stroke survivors. That consistency constitutes evidence that fear avoidance behavior is a robust construct. This might be due to similarities in living styles and level of physical activity across the Western population and Hong Kong Chinese population. As Hong Kong Chinese people is living in a multicultural environment and having westernized living styles, it is understandable that their beliefs about maintaining balance during activities and attitudes towards falls should be similar to those of the Westerners. Based on the validation of the SAFE-C with stroke survivors, the present study provides further evidence of the factor structure’s applicability. Fear avoidance behavior is a homogeneous construct that can serve as a unidimensional measure. The high consistency of the factor structure suggested that the SAFE-C is a culturally relevant and valid measure of fear avoidance behavior.

It is important to note that this study’s participants were recruited by convenience sampling and self-selected. They were relatively young and active with good functional mobility. The findings may not generalize to less able stroke survivors, and certainly not to persons not fulfilling the study’s inclusion criteria. The sample size of 20 was barely enough for assessing the test-retest reliability. Besides, details of the translation process were not reported in Chou [14] study in which the SAFE-C was first used in Chinese elderly living in nursing homes. Thus, the discussion of cultural appropriateness is limited in the present study. Note too that no confirmatory factor analysis was conducted to support the recommended 1-factor structure of the SAFE-C. Further investigation of the factor structure and testing of the SAFE-C which involves less able stroke survivors are recommended.

## Conclusions

Although the cultural appropriateness is not explored the present study, our results support the validity and reliability of the SAFE-C. It’s psychometric properties are consistent with those reported for the English version and from studies with community-dwelling older people [10] and individuals with Parkinson disease [12]. The results also suggest that the activities of community-dwelling stroke survivors are only mildly restricted by fear of falling as evidenced by the reported SAFE-C item mean scores (0.71–1.44, indicating “not all worried” to “a little worried”).” They have alternatives which they can turn to in high fall risk situations. Clinicians may choose to incorporate this instrument as their standard for assessing fear avoidance behavior among stroke survivors. The findings will also help clinicians consider the therapeutic interventions such as cognitive-behavioral therapy to reduce fear of falling and activity avoidance.

## Supporting information

S1 File This is the SAFE-C dataset file. (XLSX)

S2 File This is the scoring information guide file. (PDF)

S3 File This is the SAFE-C file. (PDF)
