# Supplementary material for: The reliability and validity of the Survey of Activities and Fear of Falling in the Elderly for assessing fear and activity avoidance among stroke survivors
Source: PLoS One. 2019 Apr 1;14(4):e0214796. doi: 10.1371/journal.pone.0214796 (PMC6443158; doi:10.1371/journal.pone.0214796)
Supplement: S2 File — (PDF) [file pone.0214796.s002.pdf]

Activity Questionnaire

A. Do you currently:	1. Go to the store?<br><br>1. NO → GO TO C<br>2. YES → GO TO B	2. Prepare simple meals?<br><br>1. NO → GO TO C<br>2. YES → GO TO B
B. When you........, how worried are you that you might fall?	1. Very worried<br>2. Somewhat worried<br>3. A little worried, or<br>4. Not at all worried<br>GO TO F	1. Very worried<br>2. Somewhat worried<br>3. A little worried, or<br>4. Not at all worried<br>GO TO F
C. Do you not [ACTIVITY] because you are......... that you might fall?	1. Very worried<br>2. Somewhat worried → GO TO D<br>3. A little worried<br>Or<br>4. Not at all worried → GO TO E	1. Very worried<br>2. Somewhat worried → GO TO D<br>3. A little worried<br>Or<br>4. Not at all worried → GO TO E
D. Are there other reasons that you do not........	1. NO<br>2. YES → SPECIFY:__________<br>____________________________<br>____________________________<br>GO TO F	1. NO<br>2. YES → SPECIFY:__________<br>____________________________<br>____________________________<br>GO TO F
E. What are the reasons that you do not........	SPECIFY:____________________<br>________________________________<br>________________________________<br>GO TO F	SPECIFY:____________________<br>________________________________<br>________________________________<br>GO TO F
F. Compared to 5 years ago, would you say that you........	1. More than you used to,<br>2. About the same, or<br>3. Less than you used to.	1. More than you used to,<br>2. About the same., or<br>3. Less than you used to.

A. Do you currently:	3. Take a tub bath?<br><br>1. NO ↓ GO TO C<br>2. YES ↓ GO TO B	4. Get out of bed?<br><br>1. NO ↓ GO TO C<br>2. YES ↓ GO TO B
B. When you........, how worried are you that you might fall?	1. Very worried<br>2. Somewhat worried<br>3. A little worried, or<br>4. Not at all worried<br>GO TO F	1. Very worried<br>2. Somewhat worried<br>3. A little worried, or<br>4. Not at all worried<br>GO TO F
C. Do you not [ACTIVITY] because you are......... that you might fall?	1. Very worried<br>2. Somewhat worried → GO TO D<br>3. A little worried<br>Or<br>4. Not at all worried → GO TO E	1. Very worried<br>2. Somewhat worried → GO TO D<br>3. A little worried<br>Or<br>4. Not at all worried → GO TO E
D. Are there other reasons that you do not........	1. NO<br>2. YES → SPECIFY:__________<br>__________________________<br>__________________________<br>GO TO F	1. NO<br>2. YES → SPECIFY:__________<br>__________________________<br>__________________________<br>GO TO F
E. What are the reasons that you do not........	SPECIFY:____________________<br>______________________________<br>______________________________<br>GO TO F	SPECIFY:____________________<br>______________________________<br>______________________________<br>GO TO F
F. Compared to 5 years ago, would you say that you........	1. More than you used to,<br>2. About the same, or<br>3. Less than you used to.	1. More than you used to,<br>2. About the same, or<br>3. Less than you used to.

A. Do you currently:	5. Take a walk for exercise?<br><br>1. NO ↓ GO TO C<br>2. YES ↓ GO TO B	6. Go out when it is slippery?<br><br>1. NO ↓ GO TO C<br>2. YES ↓ GO TO B
B. When you........, how worried are you that you might fall?	1. Very worried<br>2. Somewhat worried<br>3. A little worried, or<br>4. Not at all worried<br>GO TO F	1. Very worried<br>2. Somewhat worried<br>3. A little worried, or<br>4. Not at all worried<br>GO TO F
C. Do you not [ACTIVITY] because you are......... that you might fall?	1. Very worried<br>2. Somewhat worried → GO TO D<br>3. A little worried<br>Or<br>4. Not at all worried → GO TO E	1. Very worried<br>2. Somewhat worried → GO TO D<br>3. A little worried<br>Or<br>4. Not at all worried → GO TO E
D. Are there other reasons that you do not........	1. NO<br>2. YES → SPECIFY:__________<br>____________________<br>____________________<br>GO TO F	1. NO<br>2. YES → SPECIFY:__________<br>____________________<br>____________________<br>GO TO F
E. What are the reasons that you do not........	SPECIFY:____________________<br>____________________<br>____________________<br>GO TO F	SPECIFY:____________________<br>____________________<br>____________________<br>GO TO F
F. Compared to 5 years ago, would you say that you.....	1. More than you used to,<br>2. About the same, or<br>3. Less than you used to.	1. More than you used to,<br>2. About the same., or<br>3. Less than you used to.

A. Do you currently:	7. Visit a friend or relative?<br>1. NO ↓ GO TO C<br>2. YES ↓ GO TO B	8. Reach for something over your head?<br>1. NO ↓ GO TO C<br>2. YES ↓ GO TO B
B. When you........, how worried are you that you might fall?	1. Very worried<br>2. Somewhat worried<br>3. A little worried, or<br>4. Not at all worried<br>GO TO F	1. Very worried<br>2. Somewhat worried<br>3. A little worried, or<br>4. Not at all worried<br>GO TO F
C. Do you not [ACTIVITY] because you are......... that you might fall?	1. Very worried<br>2. Somewhat worried → GO TO D<br>3. A little worried<br>Or<br>4. Not at all worried → GO TO E	1. Very worried<br>2. Somewhat worried → GO TO D<br>3. A little worried<br>Or<br>4. Not at all worried → GO TO E
D. Are there other reasons that you do not....	1. NO<br>2. YES → SPECIFY:__________<br>__________________________<br>__________________________<br>GO TO F	1. NO<br>2. YES → SPECIFY:__________<br>__________________________<br>__________________________<br>GO TO F
E. What are the reasons that you do not........	SPECIFY:____________________<br>______________________________<br>______________________________<br>GO TO F	SPECIFY:____________________<br>______________________________<br>______________________________<br>GO TO F
F. Compared to 5 years ago, would you say that you.....	1. More than you used to,<br>2. About the same, or<br>3. Less than you used to.	1. More than you used to,<br>2. About the same., or<br>3. Less than you used to.

A. Do you currently:	9. Go to a place with crowds?<br><br>1. NO ↓ GO TO C<br>2. YES ↓ GO TO B	10. Walk several blocks outside?<br><br>1. NO ↓ GO TO C<br>2. YES ↓ GO TO B
B. When you........, how worried are you that you might fall?	1. Very worried<br>2. Somewhat worried<br>3. A little worried, or<br>4. Not at all worried<br>GO TO F	1. Very worried<br>2. Somewhat worried<br>3. A little worried, or<br>4. Not at all worried<br>GO TO F
C. Do you not [ACTIVITY] because you are......... that you might fall?	1. Very worried<br>2. Somewhat worried → GO TO D<br>3. A little worried<br>Or<br>4. Not at all worried → GO TO E	1. Very worried<br>2. Somewhat worried → GO TO D<br>3. A little worried<br>Or<br>4. Not at all worried → GO TO E
D. Are there other reasons that you do not....	1. NO<br>2. YES → SPECIFY:__________<br>____________________<br>____________________<br>GO TO F	1. NO<br>2. YES → SPECIFY:__________<br>____________________<br>____________________<br>GO TO F
E. What are the reasons that you do not........	SPECIFY:____________________<br>____________________<br>____________________<br>GO TO F	SPECIFY:____________________<br>____________________<br>____________________<br>GO TO F
F. Compared to 5 years ago, would you say that you.....	1. More than you used to,<br>2. About the same, or<br>3. Less than you used to.	1. More than you used to,<br>2. About the same., or<br>3. Less than you used to.

A. Do you currently:	11. Bend down to get something?<br><br>1. NO ↓ GO TO C 2. YES ↓ GO TO B
B. When you........, how worried are you that you might fall?	1. Very worried<br>2. Somewhat worried<br>3. A little worried, or<br>4. Not at all worried<br>GO TO F
C. Do you not [ACTIVITY] because you are......... that you might fall?	1. Very worried<br>2. Somewhat worried → GO TO D<br>3. A little worried<br>Or<br>4. Not at all worried → GO TO E
D. Are there other reasons that you do not....	1. NO<br>2. YES → SPECIFY:__________<br>___________________________<br>___________________________<br>GO TO F
E. What are the reasons that you do not........	SPECIFY:____________________<br>______________________________<br>______________________________<br>GO TO F
F. Compared to 5 years ago, would you say that you.....	1. More than you used to,<br>2. About the same, or<br>3. Less than you used to.
