# Supplementary material for: The reliability and validity of the Survey of Activities and Fear of Falling in the Elderly for assessing fear and activity avoidance among stroke survivors
Source: PLoS One. 2019 Apr 1;14(4):e0214796. doi: 10.1371/journal.pone.0214796 (PMC6443158; doi:10.1371/journal.pone.0214796)
Supplement: S3 File — (PDF) [file pone.0214796.s003.pdf]

The Survey of Activities and Fear of Falling in the Elderly
(SAFE 恐懼跌倒評估)

	(0=not at all worried沒有害怕, 1=a little worried 小小害怕, 2=somewhat worried 有點害怕, 3=very worried 十分害怕)
1. Go to the store 行商店	0 1 2 3
2. Prepare simple meals 預備用膳	0 1 2 3
3. Take a tub bath 沖浴缸浴	0 1 2 3
4. Get out of bed 起床	0 1 2 3
5. Take a walk for exercise 行路運動	0 1 2 3
6. Go out when it is slippery 路面濕滑時仍外出	0 1 2 3
7. Visit a friend or relative 探訪朋友或親戚	0 1 2 3
8. Reach for something over your head 接觸過頭的東西	0 1 2 3
9. Go to a place with crowds 去人多擠迫的地方	0 1 2 3
10. Walk several blocks outside 自行行幾條街	0 1 2 3
11. Bent down to get something 屈身執拾東西	0 1 2 3
